# Gender, time use and overweight and obesity in adults: Results of the Brazilian Longitudinal Study of Adult Health (ELSA-Brasil)

**DOI:** 10.1371/journal.pone.0194190

**Published:** 2018-03-13

**Authors:** Karina Araujo Pinto, Rosane Harter Griep, Lucia Rotenberg, Maria da Conceição Chagas Almeida, Rosane Sousa Barreto, Estela M. L. Aquino

**Affiliations:** 1 School of Nursing, Federal University of Bahia, Salvador, Bahia, Brazil; 2 Laboratory of Health and Environmental Education, Oswaldo Cruz Institute (Fiocruz), Rio de Janeiro, Rio de Janeiro, Brazil; 3 Gonçalo Moniz Institute, Fiocruz, Salvador, Bahia, Brazil; 4 Climério de Oliveira Maternity Hospital, Federal University of Bahia, Salvador, Bahia, Brazil; 5 Institute of Collective Health, Federal University of Bahia, Salvador, Bahia, Brazil; University of Tennessee Health Science Center, UNITED STATES

## Abstract

Perceived time constraints have been highlighted in sociological studies as representing a core issue in determining quality of life. The objective of this study was to test the hypothesis that gender inequalities regarding insufficiency of time play a role in the development of overweight and obesity in adults. The study used baseline data (2008–2010) from the Brazilian Longitudinal Study of Adult Health (ELSA-Brasil), which monitors a cohort of 15,105 civil servants of 35 to 74 years of age. Insufficient time for personal care and leisure due to professional and domestic duties, as detailed in a structured questionnaire, comprised the main exposure variable. The outcome variable was overweight/obesity measured according to body mass index. Prevalence ratios were calculated using multinomial logistic regression. A greater proportion of women compared to men reported insufficient time for personal care and leisure (34.5% versus 23.8%, respectively). The prevalence of overweight was greater in men, while obesity was more common in women. Insufficient time for personal care and leisure was associated with overweight (PR = 1.29; 95% CI: 1.04–1.61) and obesity (PR = 1.65; 95% CI: 1.28–2.12) only in women working over 40 hours/week. No significant association was found for males. These results suggest that the length of the working week influences factors underlying weight gain, possibly issues linked to behavior and/or stress mechanisms. The fact that such an association was restricted to women suggests that the results originate from gender inequalities involving relationships between time and health. The findings of this study provide data on which to base public policies aimed at encouraging the redistribution of domestic responsibilities in the direction of gender equity, as well as macrosocial policies such as providing public schools for workers’ children.

## Introduction

Perceived time constraints have been highlighted in sociological studies as a central issue for quality of life [1–4]. Several characteristics of the modern world account for this perception. Firstly, the increasing contingency of employees working antisocial hours in a society that demands round-the-clock production and availability of services [5]; secondly, the intrusion of work into individuals’ personal life as a consequence of information technology providing access to employees at any time of the day or night [6]; and, finally, the greater participation of women in the job market, with slower changes in the domestic sphere, creating challenges for women with respect to the demands made on them in their personal and professional life [7].

This context also encompasses the debate on the work-family conflict that arises when the roles played in these two spheres are incompatible [8], since the demands of one exhaust all the individual’s time and physical and mental energy, leaving insufficient resources for the activities of the other [9]. The majority of studies dealing with this approach have analyzed the effect of job demands on family life [10], while others have evaluated the implications in the opposite direction [11].

The dynamics of professional and family life are strongly affected by asymmetric gender relationships, involving differentiated demands for men and women [1,3,12,13]. Indeed, the pattern of inequality in the division of household chores between men and women persists and, even in developed countries, within the domestic sphere the burden remains greater for women, particularly if there are children and/or a spouse or a person who requires care [3,11]. The need to conciliate different demands within a daily process of negotiation under time constraints affects the hierarchy of women and men’s priorities, encouraging them to reduce the use of time for themselves, with a possible adverse effect on their health and on their ability to maintain healthy lifestyle habits [3,10,12,13].

Despite the broad scope of sociological literature on time use during the 24 hours of the day, even from the perspective of gender, few studies have focused on the relationship between time use and health. One of the issues that have been investigated is obesity, which has been associated both with time use [2] and with long working hours [14]. The analyses of nutritional patterns and obesity are based on the concept that, although the multi-causality of obesity has been well documented [15–17], the interaction between socio-environmental and biological factors in the occurrence of obesity remains to be fully clarified. Empirical results have shown that mechanisms involved in the increasing magnitude of the accumulation of body weight appear to develop as a function of the underlying lifestyle characteristics of individuals in current society [18–20].

From a quantitative viewpoint, time is an organizing element for the dynamics of daily life. Thus, excessive time spent on any given activity reduces the time available for other activities, including those such as physical exercise that influence health [21]. Gender differences in time distribution and their relationships with health have seldom been investigated. The hypothesis of the present study is that gender inequalities in the time available for one's own care and leisure play a role in the development of overweight and obesity in adults.

## Materials and methods

### Ethical approval and participant consent

The ELSA-Brasil study was approved by the internal review boards of the six institutions involved (*Hospital de Clínicas de Porto Alegre*, *Universidade Federal do Rio Grande do Sul*, *Universidade Federal do Espírito Santo*, *Universidade Federal de Minas Gerais*, *Universidade Federal da Bahia*, *Universidade de São Paulo* and the *Fundação Osvaldo Cruz*) and by the National Committee for Ethics in Research (CEP/CONEP system) under reference #13065. All the study procedures were conducted in compliance with Resolution 196/96 of the National Health Council in effect at that time. All participants provided written, informed consent for their clinical records to be used in this study prior to enrolment.

### Data

This study was conducted using baseline data from the Brazilian Longitudinal Study of Adult Health (ELSA-Brasil), a prospective multicenter study currently being carried out in this country with civil servants working at five federal universities and one research center, and whose detailed description has been published elsewhere [22]. In brief, the ELSA-Brasil cohort consists of 15,105 active and retired volunteers of both sexes, ranging in age from 35 to 74 years at baseline, with recruitment having taken place between 2008 and 2010. This study combined participants from three different segments of civil service: faculty members, administrative and technical staff, and support workers. To achieve better distribution, recruitment goals were defined by sex, age and occupational category. At baseline, the participants were submitted to clinical exams and face-to-face interviews. For the present study, data were analyzed from 12,066 women and men still in active employment at that time and for whom the data of interest were available.

### Exposure variable

The principal exposure variable was “*insufficient time for personal care and leisure because of job- and family-related demands*”, obtained from responses to the statement: *“Family or professional demands (requirements or requests) prevent you from using the amount of time you would like to have to take care of yourself or for leisure”*. The participants were asked to what extent they agreed with this statement, with response options being: “never or almost never”, “rarely”, “sometimes”, “often” or “very often”, rendering a score that ranged from 0 to 4 in that order. This variable was dichotomized, with scores of 3 and 4 points grouped together into a category defined as “yes” and the other scores grouped together into the reference category “no”. The item was found to have temporal stability when its psychometric proprieties were evaluated using the kappa statistic, resulting in a weighted kappa index of 0.70 (0.63–0.77) [23].

### Outcome variable

The outcome variable was *overweight/obesity*, based on the calculation of body mass index (BMI). The anthropometric measurements included weight and height. All the participants’ measurements were taken under baseline conditions consisting of 8–12 hours fasting.

Body weight was measured using Toledo^®^ scales with a capacity of up to 200 kg. Height was measured using Seca^®^ stadiometers appropriate for taking anthropometric measurements for research purposes. The equipment and their respective installation and calibration procedures were standardized for all the research centers involved in the ELSA-Brasil study [24].

Participants were classified as overweight or obese based on their BMI, calculated using the formula BMI = weight(kg)/height(m)^2^. This is the indirect measurement most commonly used and that recommended by the World Health Organization for the evaluation of overweight/obesity in adults [25]. Overweight was defined as a BMI of 25 to 29 kg/m^2^ and obesity as BMI ≥ 30 kg/m^2^. Three categories were thus defined for the outcome variable: normal weight/underweight (1), overweight (2) and obesity (3).

### Covariates

The major association was adjusted for other covariates indicated in the literature as being associated with overweight/obesity [12,16,17] and time scarcity [21]. Covariates related to the work and family domains were included in the analyses. Regarding the work domain, the responses to a summarized and adapted version of the Swedish Demand-Control-Support Questionnaire [26] were used, with the following covariates being extracted and dichotomized (0 and 1 in that order): job demands (low and high); level of control at work (high and low); and social support at work (high and low). A covariate was also included on the quantification of the working week (≤ 40 hours or > 40 hours/week).

The following covariates related to the family were selected and dichotomized (0 and 1 in that order): being in a stable marital union (“no” for those who were single or not living with a partner and “yes” for those who were married or living with a partner); presence of children (no/yes); presence of a full-time maid (yes/no) and having to care for a sick or handicapped person (no/yes).

### Data analysis

All the analyses were performed separately for women and men. Pearson’s chi-square test was used in the descriptive analysis of the exposure and outcome variables, and for all covariates, with p-values ≤ 0.05 being considered statistically significant.

Tests for ordered logistic model (using omodel command) showed that the proportional odds assumption was violated and then we opted to use a multinomial model. All covariates associated with the outcome (p≤0.05) were included in a multinomial logistic regression analysis to calculate prevalence ratios (PR) and 95% confidence intervals (95%CI), taking as a reference the classification *normal weight/underweight*. In order to identify a potential effect modifier, the magnitude of crude and adjusted associations was analyzed, together with the 95% confidence intervals. Covariates associated with the outcome (but not the exposure) and with the exposure (but not the outcome), and where in addition there was no causal link between exposure and outcome, were included in the final models for women and men. Prevalence ratios (PR) were calculated using the mlogit command of the Stata^®^ software program. The Wald test was used to verify the independence of the variables in the final models [27]. All analyses were conducted using the Stata^®^ statistical software package, version 10.

## Results

Of the 12,066 individuals analyzed in this study, 52.2% were women. The proportion of individuals reporting *insufficient time for personal care and leisure* was greater in the group of women compared to the men (34.5% and 23.8%, respectively) (Table 1 and S1 Table). Both in men and women, *insufficient time for personal care and leisure* was reported by a significantly higher proportion of workers in the 35-49-year age group, by those with higher education levels, and those self-reporting their skin color as white. All job domain variables were associated with *insufficient time for personal care and leisure*, both in the group of men and in the group of women.

**Table 1:** Proportion of individuals reporting insufficient time for personal care and leisure, according to selected variables.

Variables	Women			Men		
	n = 6,299 (52,2%)			n = 5,767 (47,8%)		
	n	%	p-value*	n	%	p-value*
**Insufficient Time for Personal Care and Leisure**	**2173**	**34.5**		**1372**	**23.8**	
**Education Level**						
Basic Education (Elementary/High School)	2797	27.4	0.000	2906	15.5	0.000
Universitary/Postgraduate courses	3502	40.1		2861	32.1	
**Age**						
35–49 years	3441	36.6	0.000	2969	25.9	0.000
≥50 years	2858	31.8		2798	21.5	
**Ethnicity**						
White	3149	37.1	0.000	2926	27.2	0.000
Black/brown/other	3150	31.8		2841	20.2	
**Job Domain:**						
**Working Week**						
≤ 40 hours	4533	28.3	0.000	3565	14.9	0.000
> 40 hours	1766	50.2		2202	38.0	
**Job Demands**						
Low	3749	26.5	0.000	3860	15.7	0.000
High	2550	46.1		1907	40.1	
**Control at Work**						
Low	3616	31.8	0.000	2941	18.3	0.000
High	2683	38.0		2826	29.4	
**Social Suport at Work**						
Low	3614	39.5	0.000	2907	30.0	0.000
High	2685	27.6		2860	17.4	
**Family Domain:**						
**In a Stable Union**						
Yes	3476	37.9	0.000	4667	24.1	0.228
No	2823	30.2		1100	22.4	
**Children**						
Yes	4801	36.9	0.000	4817	23.6	0.488
No	1498	26.6		950	24.6	
**Cares for a Sick or Handicapped Person**						
Yes	642	43.8	0.000	477	26.6	0.125
No	5656	33.4		5289	23.5	
**Presence of a full-time maid**						
Yes	1613	44.8	0.000	1280	35.5	0.000
No	4686	30.9		4486	20.4	

Cohort includes men and women who were in active employment at baseline of the ELSA-Brasil study.

ELSA-Brasil 2008–2010.

*Pearson’s X^2^

In the family domain, the greatest proportions of individuals reporting *insufficient time for personal care and leisure* were in the group of women—but not men—in a stable union, among those who had children and those who cared for a dependent individual with special needs. In the group of men, the only variable related to the family that was found to be significantly associated with having *insufficient time for personal care and leisure* was the presence of a full-time maid to do the housework (35.5%) (Table 1).

The distribution analysis of *overweight/obesity* showed a high prevalence of both overweight and obesity in both sexes, although overweight was more common in men (45.0%), while obesity was more prevalent in women (23.9%) (Table 2 and S2 Table).

**Table 2:** Prevalence of overweight and obesity, according to sex and selected variables.

	Women				Men			
Variables	n	Overweight	Obesity	p-value˚*	n	Overweight	Obesity	p-value*
		%	%			%	%	
**Overweight/Obesity**	**6,313**	**35.0**	**23.9**		**5,779**	**45.0**	**20.8**	
**Education Level**								
Basic Education (Elementary/High School)	2805	37.7	30.1	0.000	2914	44.6	22.2	0.043
Universitary/Postgraduate courses	3508	32.9	18.9		2865	45.4	19.4	
**Age**								
35–49 years	3443	32.9	21.8	0.000	2969	43.8	20.2	0.022
≥50 years	2870	37.6	26.3		2810	46.3	21.4	
**Ethnicity**								
White	3155	33.4	20.4	0.000	2928	45.0	20.3	0.511
Black/brown/other	3158	36.7	27.4		2851	45.0	21.4	
**Job Domain:**								
**Working Week**								
≤o40 hours	4539	35.6	24.1	0.145	3573	45.1	20.3	0.448
> 40 hours	1774	33.6	23.3		2206	44.9	21.6	
**Job Demands**								
Low	3749	36.1	23.8	0.095	3861	44.6	20.4	0.168
High	2564	33.5	24.0		1918	45.9	21.6	
**Control at Work**								
Low	3615	36.4	25.8	0.000	2942	44.8	21.6	0.252
High	2698	33.3	21.3		2837	45.2	20.0	
**Social Suport at Work**								
Low	3612	34.6	23.3	0.140	2906	45.2	20.9	0.903
High	2701	35.7	24.6		2873	44.8	20.7	
**Family Domain:**								
**In a Stable Union**								
Yes	3482	36.3	22.5	0.007	4676	45.4	21.3	0.012
No	2831	33.5	25.6		1103	43.4	18.8	
**Children**								
Yes	4811	36.6	24.8	0.000	4827	45.6	21.5	0.000
No	1502	30.0	20.9		952	42.2	17.5	
**Cares for a Sick or Handicapped Person**								
Yes	642	33.2	30.5	0.000	478	45.2	19.0	0.562
No	5667	35.3	23.1		5294	45.0	21.0	
**Presence of a full-time maid**								
Yes	1615	34.9	18.1	0.000	1281	46.4	20.5	0.517
No	4698	35.1	25.9		4497	44.6	20.9	

Cohort includes men and women who were in active employment at baseline of the ELSA-Brasil study.

ELSA-Brasil 2008–2010.

*Pearson’s X^2^

Women with less schooling were more likely to be obese (30.1%). Among men, the prevalence of overweight was greater in those with a university education (45.4%). Individuals of 50 years of age or more were more likely to be overweight or obese irrespective of sex. Among women, those who declared themselves black or brown-skinned were more likely to be overweight (36.7%) or obese (27.4%). This trend was not confirmed for the men (Table 2).

Among women, an association was also found between being overweight or obese and all the variables in the family domain. It is noteworthy that the greatest prevalence of overweight (36.3%) was observed in those who had children, while the greatest prevalence of obesity (30.5%) was in the group of women who cared for a sick or handicapped person. Women who were able to count on the support of a full-time maid were less likely to be obese (18.1%), while the lowest prevalence of overweight was found in the group of women with no children (30.0%) (Table 2).

Among the men who cared for a sick or disabled person and those who had a full-time maid, no statistically significant differences were found in the distribution of body weight. Those who lived with a partner and those who had children were the groups in which the prevalence rates of overweight (45.4% and 45.6%, respectively) and obesity (21.3% and 21.5%, respectively) were highest (Table 2). The highest prevalence rates of overweight (36.6%) and obesity (25.8%) were found in the group of women who reported high levels of control at work.

When selecting the covariates for inclusion in the models, the length of the working week was identified as an effect modifier in the women; therefore, stratification was made as a function of this variable. The covariates *education*, *age*, *ethnicity*, *control at work*, *children*, *caring for a sick or handicapped person* and *presence of a full-time maid* were identified as confounding factors and were included for simultaneous adjustment in the final model for the group of women. Wald’s test confirmed the independence of covariates and the good fit of the model. Having *insufficient time for personal care and leisure* was associated with overweight (PR = 1.29) and obesity (PR = 1.65) only in the group of women whose working week consisted of more than 40 hours, even following adjustment for confounding variables (Table 3).

**Table 3:** Insufficient time for personal care and leisure and overweight/obesity in women, according to working hours.

Variables	Women with a working week ≤40 hours		Women with a working week >40 hours	
	Overweight (PR^c^)	Obesity (PR^c^)	Overweight (PR^c^)	Obesity (PR^c^)
**Insufficient Time for Personal Care and Leisure**^**a**^				
No	1.00	1.00	1.00	1.00
Yes	0.86 (0.74–1.00)	0.99 (0.84–1.17)	**1.26 (1.02–1.57)**	**1.48 (1.16–1.88)**
**Insufficient Time for Personal Care and Leisure**^**b**^				
No	1.00	1.00	1.00	1.00
Yes	0.91 (0.78–1.06)	1.08 (0.91–1.28)	**1.29 (1.04–1.61)**	**1.65 (1.28–2.12)**

^a^ Unadjusted model

^b^ Model adjusted for education, age, ethnicity, control at work, children, cares for a sick or handicapped person, and presence of a full-time maid, in the group of women. ELSA-Brasil, 2008–2010.

^c^ PR: Prevalence Ratio.

In the group of men, no modifier of the principal effect was found and the covariates selected for adjustment were education, age and children (Table 4). Nevertheless, simultaneous analysis showed no evidence of any association between *insufficient time for personal care and leisure* and overweight/obesity in this group.

**Table 4:** Insufficient time for personal care and leisure and overweight/obesity in men.

	Men	
Variables	Overweight (PR^c^)	Obesity (PR^c^)
**Insufficient Time for Personal Care and Leisure**^**a**^		
No	1.00	1.00
Yes	0.97 (0.84–1.11)	1.06 (0.89–1.25)
**Insufficient Time for Personal Care and Leisure**^**b**^		
No	1.00	1.00
Yes	0.98 (0.85–1.12)	1.11 (0.94–1.32)

^a^ Unadjusted model

^b^ Model adjusted for education, age and children, in the group of men. ELSA-Brasil, 2008–2010.

^c^ PR: Prevalence Ratio.

## Discussion

Understanding the relationship between time and health in an epoch marked by the acceleration of social times is a complex challenge [21]. The results of our study indicate that the perception of not having enough time available for personal care and leisure because of household chores and family demands is associated with a greater prevalence of overweight and obesity in women whose working week consists of more than 40 hours. These findings are supported in the literature on time use in the light of gender theories, which emphasize the disadvantages for women resulting from the unequal distribution of household chores [3,12]. This study is particularly relevant since it takes into consideration the competing demands for an individual’s time from both the public and private domains, with the findings revealing the implications of time constraints resulting from excessive demands. This approach, seldom used in research studies, is similar to the “gender-sensitive” indicators mentioned in the literature [1] as relevant in highlighting the asymmetric experience of women and men in relation to time use and revealing possible social determinants of quality of life.

Studies based on time use in which participants keep a record of their activities over a certain period of time also suggest an association between temporal aspects of feeding and obesity. For instance, in a nationally representative sample of the North American population, time spent in food preparation was inversely related to BMI in women [28]. One possible mechanism involved in weight gain is that greater number of working hours (including paid and unpaid work) may be related to higher levels of stress (with higher blood cortisol rates) and, consequently, a greater likelihood of increasing food intake and/or of choosing food with a high fat and sugar content [18,20,29]. In fact, the perception of a scarcity of time may be related to preparing fewer healthy meals at home and consuming more fast food and convenience food, particularly if there is no full-time maid [29]. Experts in obesity, diet and physical activity consider time pressure as an important social trend underlying the increasing obesity rates [30].

The finding that of the women working more than 40 hours a week, those who did not have a full-time maid were more likely to be obese, should be used with caution when comparing these results directly with data in the literature. Having a full-time maid is a practice that occurs in few countries, especially in that with high gender inequality and high internal rural-urban migration and international migration. In Brazil, having women of low socioeconomic status that perform domestic chores in middle- and upper-class homes on a full-time basis is common practice [31,32]. A similar practice is observed in Malaysia [33]. Unlike the pattern of domestic service in Brazil, in some countries female immigrants are employed as full-time maids [34]. Despite the difficulty in drawing comparisons with other countries, the present findings can be considered in line with the positive association found in the Whitehall II Study between long working hours and obesity [19]. This view remits to the conception of time as a resource for good health [35], since people need time in which to practice physical activities, to visit a doctor, or to recover from work. Furthermore, it is possible that, when the individual chiefly responsible for cooking is the housemaid, then women from wealthier families who employ maids will be less involved with everyday cooking, possibly resulting in less exposure to food intake and more weight-watching.

Women with better education levels, those caring for a sick or handicapped person, and those who reported high job demands and a longer working week were those most likely to perceive time for their personal care and leisure as being insufficient, which could be interpreted as an expression of the excess burden they are subjected to in the two domains of work and family. The total number of hours worked by women, taking into consideration the time spent at work and the time spent in the domestic environment, has been reported to be a relevant factor when evaluating health outcomes in this group [36]. In other studies, researchers have also found significant gender differences with respect to the quantity and quality of free time, with women experiencing more disadvantages and greater restrictions and fragmentation of leisure time [1,7].

The approach used in the present study is innovative in Brazil and, even in the international literature few studies have opted to use body weight as the endpoint in analyses on time-based work-family conflict [37,38]. In the group of women, there was an expressive inverse association between education level and body weight, showing the important effect of schooling in reducing the prevalence of overweight and obesity in this group. In men, higher education played a significant role in decreasing the incidence of obesity; however, the frequency of overweight increased. The results of the present study are in line with previous findings from studies conducted in Brazil, including data from a large survey [39] in which education was found to be directly associated with overweight/obesity in men and inversely in women. These results may be explained in part by the arguments that better-educated women tend to value and pursue the ideal of thinness [16], even if it is a consequence of socio-cultural pressure [40], since education, social class and income are factors that are usually positively associated. Considering this relationship, higher socioeconomic status influences the need to work longer hours [14] but, on the other hand, also increases an individual’s ability to access health-promoting resources such as physical exercise programs [16,40], to live in a safe enough neighborhood to be able to exercise outside, and to have access to a greater variety of healthy foods etc.

Relatively few studies have been conducted on factors related to the family in association with overweight and obesity in adults, except those more closely associated with lifestyle such as marital status and the presence of children [11,41]. Our results confirm the findings of other studies on work-family interplay, describing how factors related to the family are more relevant for women than for men [12,36,41,42]. For the women, factors such as not having the support of a full-time maid, having to care for a sick or disabled person and having children were associated with a greater likelihood of being overweight. Having children was a factor associated with overweight and obesity in men, corroborating findings from international studies [35,43].

Although there have been reports in the international literature of an association between job-related factors (low control at work, greater demands and a greater effort-reward imbalance) and overweight/obesity [18], this was not confirmed in the present study. Only the length of the working week was found to be relevant in modifying the effect of the principal association in women. The composition of the cohort may have affected these results, since in academic life time constraints are a result of having to synchronize different demands, particularly in the group of faculty members [4].

The magnitude of the prevalence of overweight and obesity in men and women in this study merits particular attention, as it highlights the increase in these conditions compared to the findings of previous studies conducted in Brazil, reinforcing the importance of understanding the mechanisms that contribute to this occurrence [39,43,44].

The main association found here reinforces the relevant debate on the use of time and gender equity in modern times; however, due to the inherent limitations of a cross-sectional study, it is impossible to establish the temporal sequence of events with any certainty. It should be emphasized that the hypotheses tested here may be confirmed in future analyses conducted using the longitudinal data from the ELSA-Brazil study and other longitudinal studies. Notwithstanding, the ELSA-Brasil cohort is composed of civil servants who volunteered to participate in the study, which indicates the very selective nature of this study sample and, as such, any broad characterization of these results should, in principle, be interpreted with caution.

## Conclusions

These results suggest that the length of the working week influences factors underlying weight gain, possibly issues linked to behavior and/or stress mechanisms. It appears difficult for women not to become involved in domestic duties and, when such demands are combined with long working hours, the likelihood of gaining weight appears to be greater. The fact that this association was restricted to women suggests that the present findings are derived from gender inequalities involving relationships between time and health. The results of this study provide data on which to base public policies aimed at encouraging the redistribution of domestic responsibilities in the direction of gender equity, as well as macrosocial policies such as providing public schools for workers’ children.

## Supporting information

S1 Table Perception of *insufficient time for personal care and leisure*, according to sex. (DOCX)

S2 Table Distribution of body mass index (kg/m^2^), according to age groups and sex. (DOCX)
