# Correction: Gender, time use and overweight and obesity in adults: Results of the Brazilian Longitudinal Study of Adult Health (ELSA-Brasil)

**DOI:** 10.1371/journal.pone.0198380

**Published:** 2018-08-08

**Authors:** Karina Araujo Pinto, Rosane Harter Griep, Lucia Rotenberg, Maria da Conceição Chagas Almeida, Rosane Sousa Barreto, Estela M. L. Aquino

The fourth author’s name appears incorrectly in the citation. The correct citation is: Pinto KA, Griep RH, Rotenberg L, Almeida MdCC, Barreto RS, Aquino EML (2018) Gender, time use and overweight and obesity in adults: Results of the Brazilian Longitudinal Study of Adult Health (ELSA-Brasil). PLoS ONE 13(3): e0194190. https://doi.org/10.1371/journal.pone.0194190
