# Supplementary material for: Gender, time use and overweight and obesity in adults: Results of the Brazilian Longitudinal Study of Adult Health (ELSA-Brasil)
Source: PLoS One. 2018 Mar 13;13(3):e0194190. doi: 10.1371/journal.pone.0194190 (PMC5849321; doi:10.1371/journal.pone.0194190)
Supplement: S1 Table — (DOCX) [file pone.0194190.s001.docx]

Appendice 1

S1 Table. Perception of *Insufficient Time for Personal Care and Leisure*, according to sex.

|  | **WOMEN** | | **MEN** | | **TOTAL** | |
| --- | --- | --- | --- | --- | --- | --- |
|  | **n** | **%** | **n** | **%** | **n** | **%** |
| Very frequently | 608 | 9.6 | 300 | 5.2 | 908 | 7.5 |
| Frequently | 1575 | 24.8 | 1071 | 18.5 | 2646 | 21.8 |
| Sometimes | 2061 | 32.5 | 1851 | 32.0 | 3912 | 32.2 |
| Rarely | 1114 | 17.6 | 1421 | 24.6 | 2535 | 20.9 |
| Never/almost never | 986 | 15.5 | 1143 | 19.7 | 2129 | 17.6 |

Longitudinal Study of Adult Health (ELSA – BRASIL), 2008-2010.
