# Supplementary material for: Gender, time use and overweight and obesity in adults: Results of the Brazilian Longitudinal Study of Adult Health (ELSA-Brasil)
Source: PLoS One. 2018 Mar 13;13(3):e0194190. doi: 10.1371/journal.pone.0194190 (PMC5849321; doi:10.1371/journal.pone.0194190)
Supplement: S2 Table — (DOCX) [file pone.0194190.s002.docx]

Appendice 2

S2 Table. Distribution of Body Mass Index (kg/m^2^), according to age groups and sex.

| **Age groups** |  | **WOMEN** | | |  | **MEN** | | |
| --- | --- | --- | --- | --- | --- | --- | --- | --- |
|  |  | Sample size | Mean | Standard deviation |  | Sample size | Mean | Standard deviation |
| **All ages (years)** |  | 6313 | 26.88 | 5.06 |  | 5779 | 26.97 | 4.32 |
| 35-39 |  | 617 | 25.96 | 5.33 |  | 540 | 26.30 | 4.43 |
| 40-44 |  | 1158 | 26.26 | 4.93 |  | 1021 | 26.80 | 4.50 |
| 45-49 |  | 1668 | 26.79 | 5.02 |  | 1408 | 27.11 | 4.28 |
| 50-54 |  | 1440 | 27.26 | 5.06 |  | 1242 | 27.20 | 4.35 |
| 55-59 |  | 966 | 27.56 | 5.04 |  | 999 | 27.05 | 4.16 |
| 60-64 |  | 340 | 27.36 | 4.93 |  | 432 | 27.03 | 3.97 |
| 65-69 |  | 124 | 27.32 | 4.45 |  | 134 | 26.47 | 4.15 |
| 70-74 |  | 0 | - | - |  | 3 | 23.34 | 2.61 |

Longitudinal Study of Adult Health (ELSA – BRASIL) 2008-2010.
